# Aboriginal and non-aboriginal Australian former prisoners’ patterns of morbidity and risk of hospitalisation

**DOI:** 10.1186/s12939-016-0497-3

**Published:** 2017-01-05

**Authors:** Jane E. Lloyd, Elizabeth McEntyre, Eileen Baldry, Julian Trofimovos, Devon Indig, Penelope Abbott, Jennifer Reath, Kathy Malera-Bandjalan, Mark F. Harris

**Affiliations:** 1 Centre for Primary Health Care and Equity, Faculty of Medicine, University of New South Wales, Sydney, Australia; 2 School of Social Sciences, Faculty of Arts and Social Sciences, University of New South Wales, Sydney, Australia; 3 School of Public Health and Community Medicine, Faculty of Medicine, University of New South Wales, Sydney, Australia; 4 Department of General Practice, Faculty of Medicine, Western Sydney University, Sydney, Australia; 5 Aboriginal and Islander Health Worker Journal, Sydney, Australia

**Keywords:** Aboriginal Australians, Access to health care, Hospitalisation, Criminal justice

## Abstract

**Background:**

People who have been in custody are more likely to experience multiple, long standing health issues. They are at high risk of illness and injury post release and experience poor access to health services both of which contribute to high rates of recidivism. The study was conducted to examine Aboriginal and non-Aboriginal former prisoners’ risk of hospitalisation and rehospitalisation in the five years post release from custody and identified the common reasons for hospitalisations.

**Methods:**

Common reasons for hospital admission were identified by conducting descriptive analysis of linked data, related to former prisoners, from NSW Ministry of Health and Corrective Services NSW. This relied upon admitted patient data for 1899 patients. Of this cohort, 1075 people had been admitted to hospital at least once and remained out of custody over a five year period. The independent variables we studied included age, sex, and whether or not the person was Aboriginal. We conducted univariate and multivariate analysis on the following dependent variables: number of admissions over five years after release; more than one admission; days between custody and first hospitalisation; and days between first and second hospitalisation.

**Results:**

Mental and behavioural disorders, injuries and poisoning, and infectious or parasitic diseases were the three most common reasons for admission. Aboriginal and non-Aboriginal former prisoners had a broadly similar pattern of reasons for admission. Yet Aboriginal former prisoners were more likely than non-Aboriginal former prisoners to have a shorter mean interval between hospital admission and readmission (187 days compared to 259 days, t = 2.90, *p*-0.004).

**Conclusions:**

Despite poorer health among Aboriginal people, there were broadly similar patterns of reasons for admission to hospital among Aboriginal and non-Aboriginal former prisoners. There may be a number of explanations for this. The cohort was not a representative sample of the NSW prison population. There was an overrepresentation of individuals with cognitive disability (intellectual disability, acquired brain injury, dementia, fetal alcohol spectrum disorder) in the study population, which may have impacted on this group accessing hospital health care. Alternatively perhaps there were fewer presentations to hospital by Aboriginal former prisoners despite a greater need.

The shorter interval between hospital admission and readmission for Aboriginal former prisoners may suggest the need for better follow up care in the community after discharge from hospital. This presents an opportunity for primary health care services to work more closely with hospitals to identify and manage Aboriginal former prisoners discharged from hospital so as to prevent readmission.

## Background

Aboriginal people are over-represented in the Australian criminal justice system and their rate of incarceration is increasing. In the March quarter 2016, the proportion of Aboriginal prisoners represented 28% of the total full-time prisoner population, whereas Aboriginal people comprise 2% of the general Australian population aged 18 years and over. The rate of Aboriginal incarceration is increasing. Between the March quarter 2015 and the March quarter 2016 the imprisonment rate for Aboriginal Australians increased by 7% [1]. Aboriginal Australians are more likely to cycle in and out of prison on remand or by serving multiple short sentences [2, 3].

Aboriginal people who have been in custody are likely to experience multiple, long standing health issues [4], and to be at a high risk of illness and injury post release [5, 6]. In particular many Aboriginal people who have been in custody have a mental illness and/or a cognitive disability [7], a substance abuse disorder and a chronic condition [8]. Aboriginal people in custody have higher rates of mental illness than other Aboriginal Australians [9]. Aboriginal Australians have been deeply negatively affected over generations by colonisation, dispossession, having children stolen, being discriminated against and being subjected to multiple forms of institutionalisation [10]. Aboriginal Australians’ overrepresentation in the criminal justice system cannot be understood without this knowledge [11, 12].

### The impact of incarceration on health

Health services in prisons are not fully equipped to manage mental illness and other disabilities [13]. Often mental illness and anxiety and some forms of physical illness such as diabetes are hidden problems that have gone undetected prior to imprisonment and therefore stay undetected by both the prisoner and the criminal justice system in the context of more immediate concerns. This is especially true during transition into and out of prison on remand or short sentence where it becomes difficult to prioritise health among more pressing demands.

In addition to pre-existing poorer health among Aboriginal Australians entering the criminal justice system, being imprisoned can also worsen ill health although for some it is an opportunity to receive health care. Being incarcerated is another form of trauma that is likely to negatively impact one’s health and wellbeing [14]. In New South Wales (NSW), health services to prisoners are provided through the public health system, by the Justice Health and Forensic Mental Health Network. However the prison environment is not geared to deal with multi-morbidities; Corrective Services’ concerns for security and accommodating prisoners can take precedence over the health and welfare of prisoners [14].

### The impact of poor health and poor access to health care on recidivism

An important factor influencing recidivism is the level of services available for people on release from prison [15]. In Australia, a former prisoner’s access to health care and social support services such as housing varies depending on their custody classification and place of residence. Sentenced prisoners have better access to post release programs than those on remand [5] and short sentence prisoners (under one year) have little access to services compared with those on longer sentences [12]. Aboriginal people are likely to have more short sentences and remand episodes than non-Aboriginal people [3] and so have poorer access to and use of in-prison services. Without access to comprehensive health care and social support services, Aboriginal people are more likely to return to the same environments that led to their incarceration in the first place, thus contributing to higher rates of recidivism and hospitalisation. Particular concerns include the poor ongoing management of mental illness and substance misuse, experiences of trauma and marginalisation and difficulty securing housing [16] and employment. Accessing culturally safe primary health care [17] that deals with trauma, drug and alcohol, family violence and breakdown [18] on release is necessary to support former Aboriginal prisoners to stay out of the criminal justice system.

To date research on the hospitalisation of former prisoners has tended to focus on the immediate period after release from custody and the need for immediate transition support [6, 19]. Access to quality in-custody health care and to immediate post release care for Aboriginal Australians is important. So too is what happens in the five years post release particularly the extent to which institutions such as hospitals and primary health care services meet the needs specific of Aboriginal Australians. The aim of this study is to identify Aboriginal and non-Aboriginal ex-prisoners’ risk of hospitalisation and rehospitalisation in the five years post release from custody and to detect the common reasons for hospitalisations.

## Methods

This primary health care and criminal justice project involved a partnership between the University of New South Wales, the Aboriginal Medical Service Western Sydney, Western Sydney University, the University of Technology Sydney and the Justice Health & Forensic Mental Health Network. The project was designed with input from Aboriginal and non-Aboriginal chief investigators. The research team included academics, primary health care providers, policy officers, an Aboriginal mental health social worker and a criminologist. This mixed method study had three phases: a systematic review, in-depth interviews and a linked data set analysis. This paper reports on the findings from the linked data set analysis.

One of the chief investigators (EB) had previously developed a Mental Health Disorders and Cognitive Disability (MHDCD) linked dataset. This includes a cohort of 2731 people (673 of whom are Aboriginal) drawn from the 2001 NSW Inmate Health Survey and from the NSW Department of Corrective Services Disability Service Database, and links all available de-identified administrative records from Criminal Justice and Human Service agencies in NSW. These agencies include Corrective Services NSW, Juvenile Justice NSW, the NSW Police Force, Justice Health & Forensic Mental Health Network, the NSW Bureau of Crime Statistics and Research (BOCSAR), Legal Aid NSW, Family & Community Services, Ageing Disability and Home Care, Housing NSW and NSW Ministry of Health. Analysing information from the linked data set received ethics approval in 2006 from the Aboriginal Health and Medical Research Council of NSW (People with Mental Health Disorders and Cognitive Disability in the Criminal Justice System 569/06).

Linked data from the Admitted Patient Data Collection from the NSW Ministry of Health and Corrective Services NSW were analysed to identify the major reasons for hospitalisation (according to all the ICD-10 diagnosis codes in the reason for admission) and to better understand Aboriginal and non-Aboriginal former prisoners’ risk of hospitalisation and rehospitalisation.

We examined a five-year period post release from custody to increase understanding of the ongoing health of former prisoners and to identify whether the experiences of Aboriginal former prisoners were different to those of non-Aboriginal former prisoners over the same time period.

**Fig. 1:**
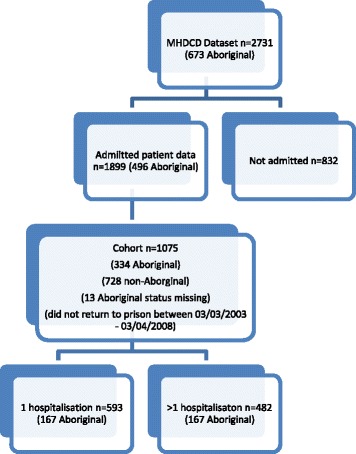
The study sample flow diagram by former prisoners’ risk of hospitalisation and rehospitalisation

Of the 2731 people in the cohort, the dataset contained admitted patient data for 1899 patients. When examining the reasons for hospital admission we utilised data from the 1899 patients. However when examining the risk of hospitalisation only 1075 of the 1899 patients fitted our inclusion criteria of remaining out of custody and having at least one hospitalisation over a five-year period from the 3rd April 2003 to the 3rd April 2008. This time period represents five years prior to the date when data was extracted from the administrative data sources. Cohort members who were re-incarcerated were excluded from the analysis so that everyone had an equal chance of being hospitalised (Fig. 1)

.

Descriptive analysis was conducted on reasons for hospitalisation. This was presented in graphic form for the three most common classes of condition (according to ICD-10 categories) for hospital admission. We then analysed factors associated with hospital admission (0 = only one hospitalisation, 1 = more than one hospitalisation). The independent variables were age (1 = 35 years and under, 2 = 36 years and over), sex (1 = male, 2 = female), and whether or not the person was Aboriginal (1 = Aboriginal, 0 = not Aboriginal). The age over 35 was chosen as the cut off for the categorisation because the incidence of premature cardiovascular disease increases from age 35 among Aboriginal people. We conducted univariate and multivariate analysis on the following dependent variables: number of admissions over 5 years after release (regression); more than one hospital admission (logistic regression); days between custody and first hospitalisation (poisson regression); and days between first and second hospitalisation (poisson regression). The analysis identified patterns of morbidity and ex-prisoners’ risk of hospitalisation post release from custody, and in particular identified similarities and differences between Aboriginal and non-Aboriginal participants.

## Results

The results are divided into two sections: Reasons for hospitalisations, and the Frequency of hospitalisation in the five years post release from custody. The first section includes all former prisoners with hospital admission data (whether they are re-incarcerated or not) and the second section is only for those who have not been re-incarcerated over the five year period specified.

### Reasons for hospitalisations

The three most common ICD-10 categories of reasons for hospitalisation were mental and behavioural disorders, injuries and poisoning and certain other consequences of external causes, and infectious or parasitic diseases. The frequencies of the top four specific diagnoses in these categories are listed in Table 1.

**Table 1:** Frequency distribution table that presents the most frequent reasons for hospitalisation in three main ICD-10 categories

	Non Aboriginal (*n* = 1403)		Aboriginal (*n* = 496)	
	N	Ratio of admission per head of population^a^	N	Ratio of admission per head of population^a^
Mental and Behavioural Disorders				
Mental and behavioural disorders due to psychoactive substance use	855	0.61	367	0.74
Schizophrenia, schizotypal and delusional disorders	285	0.20	123	0.25
Disorders of adult personality and behaviour	268	0.19	107	0.22
Neurotic, stress-related and somatoform disorders	259	0.18	96	0.19
Injury, poisoning and certain other consequences of external causes				
Injuries to the head	367	0.27	152	0.31
Poisoning by drugs, medicaments and biological substances	256	0.18	121	0.24
Injuries to the wrist and hand	241	0.17	97	0.20
Injuries to the elbow and forearm	152	0.11	65	0.13
Infectious and Parasitic diseases				
Bacterial, viral and other infectious agents	188	0.18	113	0.23
Viral hepatitis	181	0.17	66	0.13
Other bacterial diseases	36	0.03	26	0.05
Intestinal infectious diseases	27	0.03	10	0.02

^a^ (frequency of the different ICD-10 diagnoses)/number of individuals in database for whom we had admitted patient data (1403 non-Aboriginal and 496 Aboriginal = 1899)

The mean number of ICD-10 diagnosis codes per admission was 4.98. There were no significant differences in the number by Aboriginality. Instead Aboriginal and non-Aboriginal former prisoners had broadly similar reasons for admission to hospital. Females had more diagnosis codes than males (mean 5.62 compared to 4.87, *p* < 0.01) and those age more than 35 years had more morbidities than those 35 years or younger (mean 5.57 compared to 4.60, *p* < 0.001). This was confirmed by multivariate linear regression modelling.

While there were similar reasons for admission to hospital among former prisoners, Aboriginal participants had higher rates of infectious diseases caused by bacteria, viruses and other infectious agents than non-Aboriginal former prisoners (23% versus 18%).

### Multi-morbidity

**Fig. 2:**
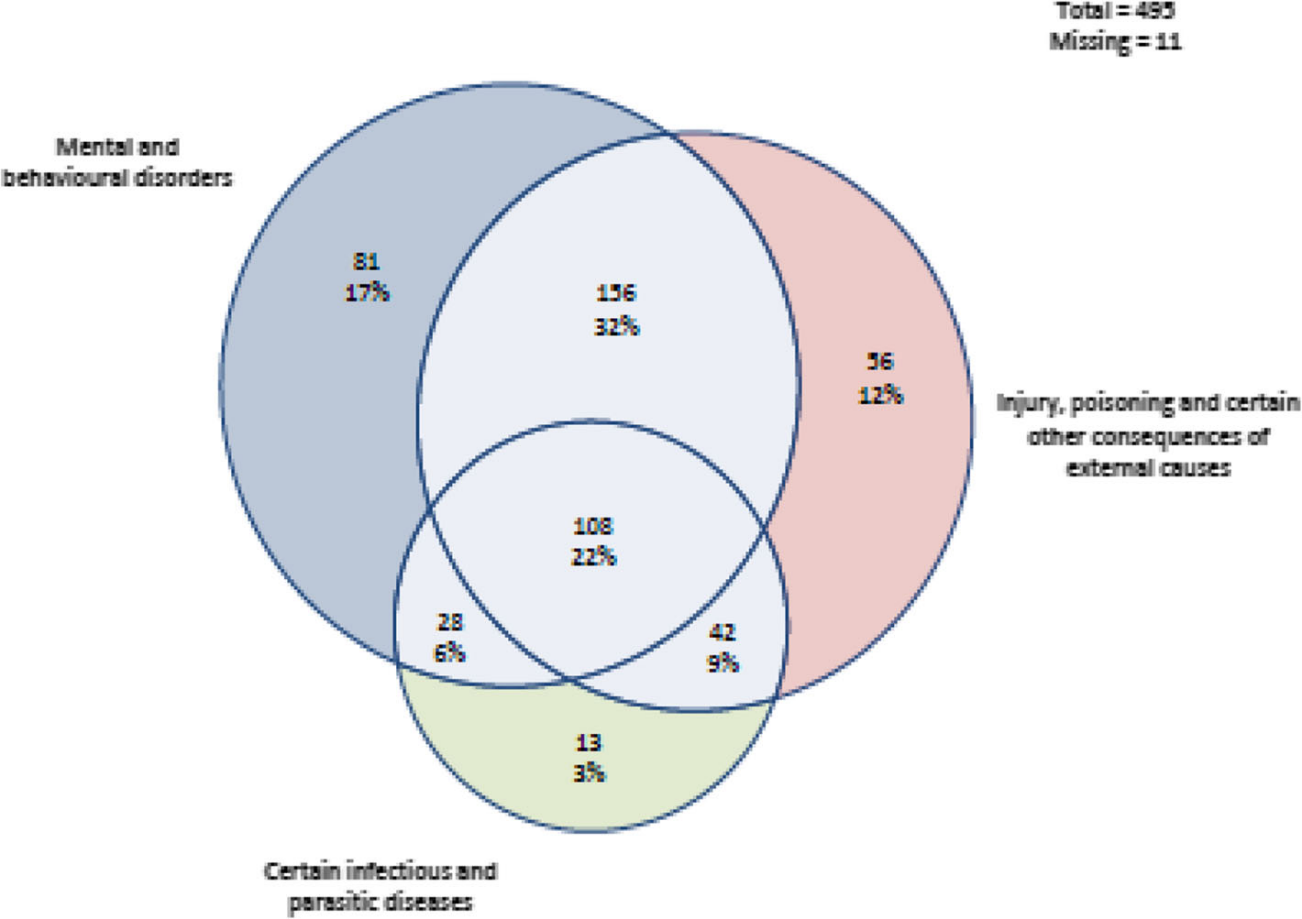
Common reasons for admission for Aboriginal Australians who had been in custody

**Fig. 3:**
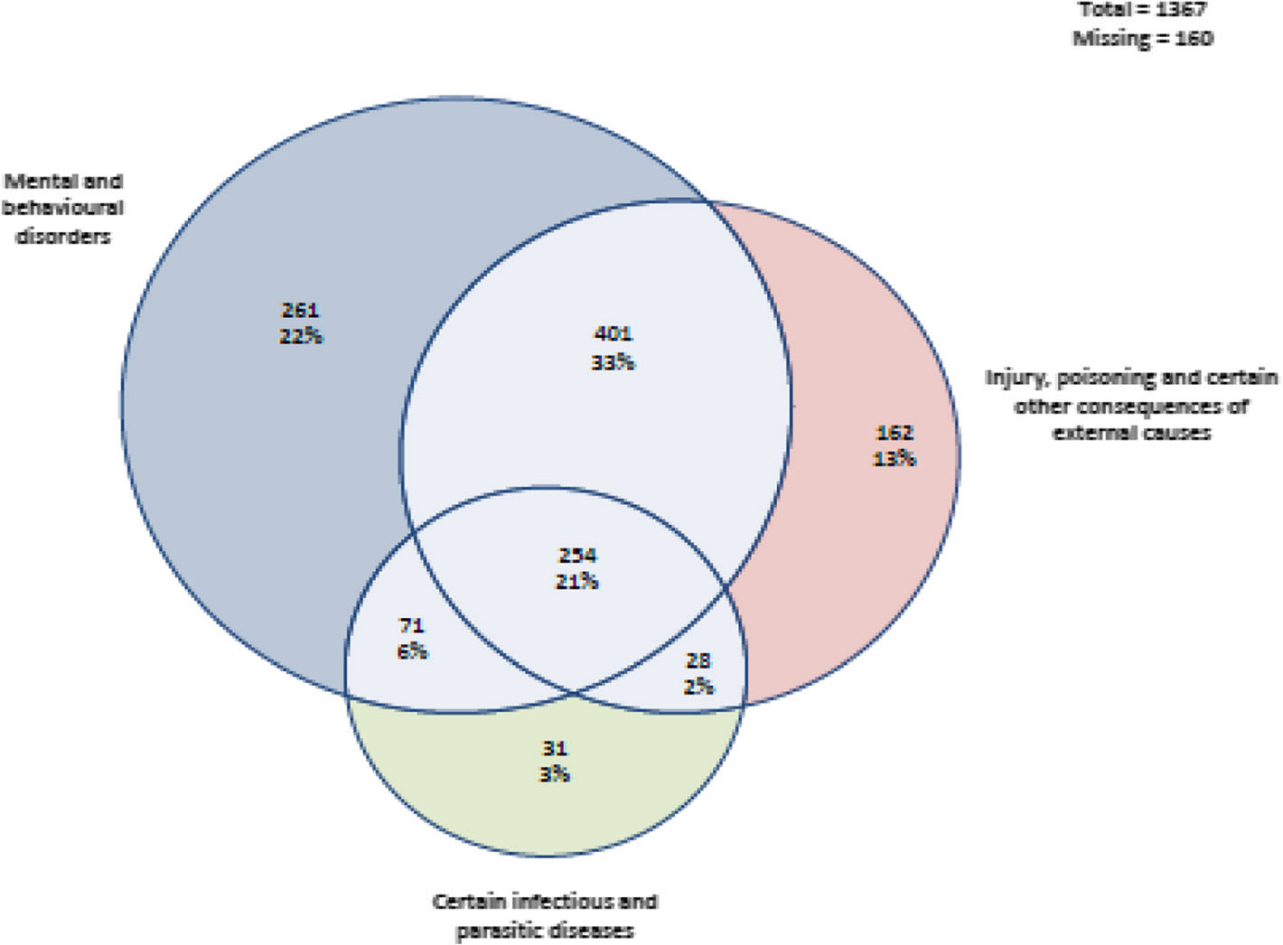
Common reasons for admission for non- Aboriginal Australians who had been in custody

Figure 2 shows the proportion of admissions for Aboriginal people that related to one or more of the three categories of diagnosis. Figure 3 shows the proportion of admissions for non-Aboriginal people that related to one or more of the three categories of diagnosis. The pattern was similar for Aboriginal and non-Aboriginal former prisoners. However, more non-Aboriginal than Aboriginal admissions were for mental and behavioural disorders alone (22% vs 17%). By contrast more Aboriginal (9%) than non-Aboriginal admissions (2%) were for both injury/poisoning and infectious disease.

### Frequency of hospitalisation

There were 1075 people in the dataset with one or more hospitalisations who did not return to prison over the five year period (April 2003- April 2008). Of these 593 people (55.2%) had only one hospitalisation and 482 (44.8%) had more than one hospitalisation. The mean number of hospitalisations over the 5 year period was 1.98. The mean age of former prisoners with only one hospitalisation was 34.9 years (SD 9.6), and the mean age of former prisoners with more than one hospitalisation was 33.1 years (SD 7.2).

A cohort of 476 met the criteria of having more than one hospitalisation in the five year period after release from custody. Among those who had more than one hospitalisation, 394 were males (81.7%) and 88 were females (18.3%), and 167 identified as being Aboriginal Australians (35.1%). For 13 cases in the cohort of 1075 information on Aboriginal status was missing. See Table 2 below:

**Table 2:** Frequency distribution table that presents the differences between Aboriginal and non-Aboriginal former prisoners admitted to hospital more than once

Hospitalisations in the five-year period	Aboriginal Australians (*N* = 334)	Non-Aboriginal Australians (*N* = 728)
Once	167 (50%)	419 (58%)
More than once	167 (50%)	309 (42%)

There was a significant difference in the likelihood of hospitalisation on more than one occasion by gender (females 56% versus males 43% *p* = 0.03). Aboriginal former prisoners were slightly more likely to be in hospital more than once compared to non-Aboriginal former prisoners (50% versus 42% *p* = 0.03). Females, younger individuals and Aboriginal Australians were more likely to be admitted to hospital more than once compared to males, older individuals and non-Aboriginal former prisoners.

In multivariate logistic regression analysis the likelihood of admission more than once was not influenced by Aboriginality after adjusting for gender and age group (Table 3). However the association for gender and age remained significant.

**Table 3:** Multivariate logistic regression analysis: Factors associated with more than one hospitalisation

Variable	Odds Ratio (95% CI)	Significance *	B	S.E
Aboriginal Australians	0.79 (0.53-1.06)	0.09	-.230	.135
Female gender	1.59 (1.25-1.94)	0.009*	.465	.177
Age group >35	0.98 (0.96-0.99)	0.001*	-.025	.007

*means that the *p* value is less than or equal to 0.05

### Interval between release from custody and first hospitalisation

The mean interval between release from custody and first hospitalisation was 353 days. This was significantly longer for those over 35 years of age (382 days) compared with those 35 and under (334 days; *p* = 0.02). There were no significant differences between Aboriginal and non-Aboriginal former prisoners or between male and female former prisoners for time to first hospitalisation. This was confirmed in generalised linear modelling adjusted for age group, sex and Aboriginality.

### Interval between hospitalisation and rehospitalisation

The mean interval between first and second hospitalisation was 239 days. The median was 71 days. For Aboriginal former prisoners the mean was 187 days compared to 259 days for non-Aboriginal former prisoners (t = 2.90, p-0.004). There was no significant difference for males and females, however older prisoners aged 35 years or more had a longer interval (t = 3.5, *p* < 0.001) than younger former prisoners. Aboriginality remained significant after adjustment for age and sex in regression analysis (Table 4).

**Table 4:** Poisson regression to identify factors associated with the number of days between first and second hospitalisation

Variable	Beta coefficient (St Error)	Significance *
Aboriginal Australians	-0.30 (0.13)	0.03 *
Male gender	-0.18 (0.13)	.17
Age group >35	0.40 (0.11)	0.001*

*means that the *p* value is less than or equal to 0.05

## Discussion

### Reasons for hospitalisation

While the reasons for hospitalisation among former prisoners were broadly similar, there were some notable differences between Aboriginal and non-Aboriginal former prisoners. More Aboriginal than non-Aboriginal former prisoners’ admissions to hospital were for both injury/poisoning and infectious disease than non-Aboriginal admissions (9% versus 2%). This suggests that Aboriginal former prisoners are more likely to have multiple conditions, thus making their health care more complex to manage. Aboriginal former prisoners had higher rates of hospitalisation for bacterial, viral and other infections than non-Aboriginal former prisoners (23% versus 18%). With early and effective management these disease hospitalisations can often be prevented. This finding suggests that Aboriginal former prisoners may delay seeking health care until it becomes absolutely necessary or are unable to access health care.

Inequitable access to health services for Aboriginal Australians [20, 21] may also explain the finding of broadly similar reasons for admissions to hospital among Aboriginal and non-Aboriginal former prisoners despite the poorer health of Aboriginal people. Not presenting to hospitals does not necessarily mean populations are well. It is possible that Aboriginal former prisoners may be less likely to present to hospital despite a greater need.

Poor access to health care and cycling in and out of prison may be part of a lifetime of institutional recycling fuelled by exclusion from continuity of appropriate support and care in schools, prisons, hospitals or other mainstream institutions [16, 22]. Whilst this exclusion may not be intentional, it is a consequence of failing to understand and address the particular circumstances and needs of Aboriginal Australians [23].

### The interval between the first and second admission to hospital

We found that Aboriginal former prisoners with a hospital admission over a five year period are more likely to have a shorter interval between the first and second admission to hospital. For Aboriginal former prisoners the mean was 187 days compared to 259 days for non-Aboriginal former prisoners (t = 2.90, *p*-0.004). This may be because Aboriginal former prisoners’ initial problems were not dealt with effectively either in hospital or after hospital discharge in the community and that Aboriginal former prisoners had poorer access to health care due to geographical and socio-economic reasons [7]. This suggests a need to ensure access to effective care by facilitating connections with primary care providers who would help the person manage their health, in order to prevent rehospitalisation and that this should be actively considered and facilitated at times of leaving prison and hospital.

### Linkage and coordination between services

The shorter interval between hospital admission and readmission for Aboriginal former prisoners may indicate a failure of hospital follow-up care and also presents an opportunity for primary health care to do more to actively support Aboriginal former prisoners discharged from hospital who may be vulnerable to discontinuity of care [24].

For Aboriginal Australians primary health care includes medical and health services provided in the community either through Aboriginal Community Controlled Health Services or through private general practice [25]. The role of primary health care providers extends beyond medical care to include recognition of the social determinants of health such as housing and employment and the need to work with other professionals and organisations to address these needs [26].

If patients are not adequately followed up and managed by primary health care providers then their conditions are unlikely to be adequately controlled, resulting in readmission to hospital. More vigorous support and referral on release from prison might assist to link Aboriginal ex-prisoners better to primary health care systems and reduce hospitalisations in the first place. Active follow-up and communication post discharge from hospital is required both with patients and their primary care provider to ensure that the provider is aware of the patient’s health status and is able to make a plan for the patient’s follow-up and ongoing care. A study of presentations to Emergency Departments in Victoria found that Aboriginal Australians were less likely to nominate a general practitioner [21], therefore if patients do not regularly see a provider, this needs to be suggested and arranged if the person so wishes.

### Complex health issues

Aboriginal former prisoners are likely to suffer complex health issues [23] and face numerous barriers to accessing culturally appropriate primary health care. Adequate long term support for Aboriginal former prisoners is likely to help prevent hospital admissions and re-incarceration. Access to good case management, culturally appropriate and continuous health care and family support are likely to reduce incarceration rates and to improve health [8, 22, 27, 28]. However there is a difference between knowing what is required in order to support Aboriginal former prisoners, and the reality of delivering such intensive and long term services. Health and social support services need to be person focused rather than disease or condition focused and are likely to require a ‘wrap around’ approach that includes services such as housing, life skills, parenting skills, mental health services, drug and alcohol and disability support [15, 29]. Primary health care is well placed to coordinate access to such services, but needs adequate infrastructure and funding in order to do so [14]. Place is another vital factor in Aboriginal former prisoners being able to access and receive appropriate health care as Aboriginal people are more highly concentrated in disadvantaged suburbs and rural areas.

The cohort used in this research was not a representative sample of the NSW prisoner population. Instead the cohort included an overrepresentation of individuals with cognitive disability. Therefore a limitation of this study is that the rates of cognitive disability are likely to be higher in this cohort than the general former prisoner population. This may have influenced the rate of admission for mental and behavioural disorders. Significance testing was not done on the reasons for hospitalisation as depicted in the Venn diagrams and therefore these findings should be treated with caution. A major limitation is also that the study was limited to people who remained out of custody over a five year period. The health issues of people who cycle in and out of prison (of which Aboriginal people are significantly over-represented) would be worse, including potentially more hospital admissions when they are in custody. This suggests these findings are an underestimate of the morbidity and complexity of the prisoner population as a whole.

## Conclusions

Despite poorer health among Aboriginal people there were broadly similar patterns of reasons for admission to hospital among Aboriginal and non-Aboriginal former prisoners.

Aboriginal Australians released from custody and who were not re-incarcerated over a five year period after release, were more likely to be readmitted to hospital in a shorter interval in the five years post release compared to their non-Aboriginal peers. The cycle of rehospitalisation may be remediated through active follow up in the community both during transition from prison as well as on release from hospital. A more proactive approach to improving access to comprehensive, appropriate and quality primary health care in the community for Aboriginal former prisoners provides the foundation for ongoing management and prevention of chronic conditions amongst this at risk group.

Adopting a longer term view of access to health care for Aboriginal former prisoners - rather than just focussing on the immediate period after release from custody - may be important in preventing a revolving door of hospitalisation.

## Abbreviations

BOCSAR: NSW bureau of crime statistics and research
ICD-10: International classification of diseases – tenth edition
MHDCD: Mental health disorders and cognitive disability
NSW: New South Wales
